# Electroencephalographic Abnormalities in Autism Spectrum Disorder: Characteristics and Therapeutic Implications

**DOI:** 10.3390/medicina56090419

**Published:** 2020-08-19

**Authors:** Francesco Precenzano, Lucia Parisi, Valentina Lanzara, Luigi Vetri, Francesca Felicia Operto, Grazia Maria Giovanna Pastorino, Maria Ruberto, Giovanni Messina, Maria Cristina Risoleo, Claudia Santoro, Ilaria Bitetti, Rosa Marotta

**Affiliations:** 1 Epilepsy and EEG lab for Developmental Age; Clinic of Child and Adolescent Neuropsychiatry, Department of Mental Health, Physical and Preventive Medicine, University of Campania “Luigi Vanvitelli”, 80138 Napoli, Italy; f.precenzano@hotmail.it (F.P.); valelanz87@hotmail.it (V.L.); mariacristinarisoleo@yahoo.it (M.C.R.); dr.claudiasantoro@gmail.com (C.S.); ilaria.bitetti@gmail.com (I.B.); 2 Inter-University Group for Study and Research on Neurodevelopmental Disorders in Children and Adolescents; lucia.parisi@unipa.it (L.P.); graziapastorino@gmail.com (G.M.G.P.); 3 Department of Psychology, Educational Science and Human Movement, University of Palermo, 90127 Palermo, Italy; 4 Department of Sciences for Health Promotion and Mother and Child Care “G. D’Alessandro”, University of Palermo, 90127 Palermo, Italy; 5 Child and Adolescent Neuropsychiatry Unit, Department of Medicine, Surgery and Dentistry, University of Salerno, 84084 Fisciano, Italy; opertofrancesca@gmail.com; 6 Centro Pro Juventute Minerva SRL, 80131 Napoli, Italy; maria.ruberto@unicampania.it; 7 Department of Clinical and Experimental Medicine, University of Foggia, 71122 Foggia, Italy; giovanni.messina@unifg.it; 8 Department of Medical and Surgical Science, University “Magna Graecia”, 88100 Catanzaro, Italy; marotta@unicz.it

**Keywords:** autism spectrum disorders, epilepsy, electroencephalogram, epileptogenic abnormalities, non-epileptiform abnormalities

## Abstract

A large body of literature reports the higher prevalence of epilepsy in subjects with Autism Spectrum Disorder (ASD) compared to the general population. Similarly, several studies report an increased rate of Subclinical Electroencephalographic Abnormalities (SEAs) in seizure-free patients with *ASD* rather than healthy controls, although with varying percentages. SEAs include both several epileptiform discharges and different non-epileptiform electroencephalographic abnormalities. They are more frequently associated with lower intellectual functioning, more serious dysfunctional behaviors, and they are often sign of severer forms of autism. However, SEAs clinical implications remain controversial, and they could represent an epiphenomenon of the neurochemical alterations of autism etiology. This paper provides an overview of the major research findings with two main purposes: to better delineate the state-of-the-art about EEG abnormalities in ASD and to find evidence for or against appropriateness of SEAs pharmacological treatment in ASD.

## 1. Introduction

The association between epilepsy and autism has long been known. Already in the first clinical descriptions of autism, Kanner reported that in a certain number of patients with autism epilepsy was also observed [1].

Autism spectrum disorder (ASD) is characterized by changes of neurological functions leading to typical neurodevelopment alterations with consequent deficits in socialization, communication, and the presence of restricted and repetitive behaviors [2].

A large body of literature reports the higher prevalence of epilepsy in subjects with ASD compared to the general population. From literature data, the prevalence of epilepsy in patients with ASD widely varies from 2% [3] to 46% [4]. The largest study focusing on the comorbidity between epilepsy and autism included nearly 6000 patients from a pre-existing research database and reported the presence of epilepsy in 12.5% of patients under 17 years old [5], whereas in a previous report on 1000 patients with ASD, about 37% showed epilepsy [6].

The presence of epilepsy also correlates with more severe autism symptoms as reported by Shubrata et al. with deficits in imitation (*p* = 0.011) and hearing domains (*p* = 0.018). The same authors also reported that a history of regression and loss of acquired skills seems to be more frequent in epileptic ASD patients (36%) than in patients without epilepsy [7].

Moreover, the prevalence of epilepsy is higher in patients with a secondary or syndromic autism form, such as tuberous sclerosis, fragile X syndrome, and other metabolic or neurological abnormalities [8,9] and in presence of intellectual disability [3,10]. Regarding the age of onset, two peaks of seizure emergence are reported: the first in early childhood and the second in adolescence [11,12,13].

There are less consistent literature data about patients with ASD and subclinical electroencephalographic abnormalities (SEAs) with absence seizures. The epileptiform activity seems to regard at least 30% of seizure-free subjects with ASD while non-epileptiform abnormalities probably seem to regard a bigger proportion of patients [14].

The reason of a so strong association between ASD, epilepsy, and electroencephalographic (EEG) anomalies can probably be found in common neuroanatomical and neurochemical events leading to a disrupted excitatory/inhibitory balance [15].

Recent evidence has demonstrated that a large number of genes are implicated in the etiopathogenesis of ASD and epilepsy expressing their influence on different aspects of excitation/inhibition balance and neuronal function such as managing synaptic vesicle release, regulation of ion channels, synaptic physiology, membrane depolarization, control of subcellular signaling pathways, and regulation of migration and organization of network connections [16].

Different types SEAs are reported in literature affecting patients with ASD: They are both non-epileptiform abnormalities such as asymmetry, slowing, and bioelectric immaturity and epileptiform abnormalities such as spikes, sharp waves, spike-and-wave, and polyspikes. SEAs are frequently seen among patients with active epilepsy psychiatric disorders, but they are rarely reported (1–4%) also in the general population of healthy children [17,18].

The presence of frequent epileptiform discharges correlates with a progressive cognitive deterioration, which is believed to be mainly due to the interference that epileptiform activity creates in normal neuronal physiology, causing a transient interruption of cognitive processes, such as plasticity, memory and language processing [19,20,21].

The aim of this narrative review was to collect an overview of original articles about the correlation between ASD and SEAs. This review helps to better delineate the state-of-the-art of main research findings about EEG abnormalities in ASD and to suggest what different SEAs provoke in subpopulations of patients with ASD. A secondary goal of this study was to find if there is solid evidence for or against treatment of SEAs in ASD.

To this end, over one hundred articles published over the years were reviewed by performing a search using the following syntax (autism OR autism spectrum disorder OR Asperger syndrome OR pervasive developmental disorders [Title/Abstract]) AND (EEG abnormalities OR EEG abnormalities OR EEG finding OR epileptiform abnormalities [Title/Abstract]). References were identified through electronic database searching in Medline (Ovid, 1946 to present) and EMBASE (Ovid), and they were adapted for Scopus (Elsevier), ERIC (Proquest), PubMed, and the Web of Science (ISI). The final database search was run on March 2020.

Abstracts were screened in order to identify only those papers that are relevant to our study.

## 2. SEAs in Non-Epileptic Patients with ASD

Many reports have shown the presence of SEAs in ASD with estimates widely varying from 4% to 86% even in the absence of a clinical history of epileptic seizures [16,22,23,24].

However, SEAs in patients with ASD exceed those of the general population (ranges from 2% to 8.7%), and this seems to be regardless of age or gender in some studies while in others they seem to decrease with puberty [25,26,27,28].

Several studies have reported SEAs in children with ASD without clinical seizures underlying that they are not signs of epilepsy but rather signs of cerebral dysfunction. (Table 1).

The most common site of EEG abnormalities was the right temporal lobe (21.5%), followed by central and bilateral temporal regions (20.2%), but parasagittal peaks were also detected in the frontal and occipital sites [30,33]. The persistence of epileptiform activity mainly in charge of the temporal lobe, as reported in several studies [34,40], suggests that the right temporal region is potentially implicated in social deficits, while bilateral and left temporal areas would correspond to the sites potentially involved in language dysfunction [30,41].

Rossi et al. found an incidence of 18.9% of SEAs in children and adults with autism, who had no previous history of epilepsy [29]. Nevertheless, Hrdlicka et al. found that 48% of SEAs (18% of non-epileptiform abnormality and 30% of epileptiform discharges) in children with ASD and without epilepsy was significantly associated (*p* = 0.014) with abnormal development during the first year of life (that is delay of language, delay in motor milestones, abnormality in social or emotional responsiveness, and behavioral difficulties), while epilepsy is correlated to intellectual disability (*p* = 0.001) evaluated through Gesell Developmental Scales and Stanford–Binet Intelligence Scale, 4th Edition [31].

In a large retrospective study, 889 patients with autism (average age: 5.3 years) with no previous history of epilepsy underwent a 24-h EEG registration for a period of 10 years. The incidence of SEAs in this population was 60.7%. Interestingly, all these patients had SEAs only in sleep. Hence the need to perform EEG recordings in sleep for adequate monitoring. The main anomalies found were spikes, sharp-waves, slow waves, generalized spike-wave complexes, polyspikes, or paradoxical delta activity [30].

More recently, Mulligan and Trauner performed a 24-h EEG recording in 101 children with autism (average age: 7.1 years). Of these, 59.4% had epileptiform discharges to EEG, and 21.8% had non-epileptiform anomalies, mainly represented by a slowdown in background activity. When only the children without a history of epilepsy were considered, 50% of these had epileptiform anomalies. Of these patients, 60% had abnormalities only during sleep, and 3.6% had epileptiform activity only in wakefulness, highlighting once again the fact that an EEG sleep is essential to detect SEAs [23,38].

The presence of epileptiform abnormalities has also been associated with lower intellectual functioning and with more serious dysfunctional behaviors [42]. On the other hand, patients classified as having high functioning autism had an incidence of only 20% of these anomalies. The fact that these patients, unlike other autistic patients, achieve good language skills, suggests that epileptiform abnormalities could have a negative effect on language development or could reflect a severer brain dysfunction, which affects language and neuronal excitability [23,43,44,45]. Moreover, patients with autism and severe intellectual disability had a higher rate of EEG abnormalities (*p* = 0.03) compared to patients with autism and mild, moderate or without intellectual disability. However, the severity of intellectual disability is not associated with abnormal MRI [46].

Interestingly, there is much evidence that SEAs are not statistically correlated to regression rate [8,32,35].

Furthermore, a study conducted on 57 patients between 2 and 18 years with the diagnosis of ASD reported (*p* = 0.047) that in seizure-free patients with epileptiform EEG anomalies, the scores of activity levels at the Childhood Autism Rating Scale (CARS) are greater compared to patients with ASD without epileptiform abnormalities [47]. Moreover, patients with epileptiform abnormalities also have a higher incidence of motor stereotypies (61% vs. 36% with or without epileptiform anomalies), a marked association of aggressive behaviors (75% vs. 0%—*p* > 0.05), and worse motor skills (*p* < 0.05) than patients without SEAs [29].

All the above studies, although with different percentages, seem to indicate that SEAs, even without clinical seizures, can cause permanent negative effects on the brain development, leading to more serious cognitive and/or behavioral deficits [37,48] and provoking severer forms of autism [39].

For instance, Capal et al. in their retrospective study found evidence that patients with ASD and SEAs exhibited more impaired adaptive functioning (evaluated through Vineland Adaptive Behavior Scales) compared with normal EEG-ASD group (*p* = 0.05) [37].

Similarly, another retrospective study by Nicotera et al. found worst levels of hyperactivity (*p* < 0.01), aggressive behaviors (*p* < 0.01), self-harm behavior (*p* < 0.01), severer IQ deficits (*p* = 0.04), and worse language abilities (*p* < 0.01) in subjects with ASD and SEAs compared to patients with ASD alone (the authors used Wechsler Scales, Griffiths mental development scales, Leiter-R scale, Language Evaluation Test—TVL, Test for Reception of Grammar TROG-2, Peabody, Mc Arthur Questionnaire, Autism Diagnostic Interview-Revised—ADI-R, Autism Diagnostic Observation Schedule Second Edition—ADOS-2, and Childhood Autism Rating Scale—CARS-2, for their evaluations) [39].

However, there are some contrasting studies that do not find statistically significant differences in behaviors in children with ASD and EEG abnormalities compared to children with ASD and normal EEG [36].

## 3. Electroencephalographic Patterns

It is very unlikely that ASD has a distinctive EEG pattern because heterogeneity is a hallmark in its etiology, phenotype, and outcome. However, epileptiform discharges seem to be more common than non-epileptiform abnormalities in EEG of patients with ASD. Epileptiform abnormalities include generalized, focal, multi-focal, unilateral, or bilateral discharges, and they are localized in different brain areas, but they are probably more common in temporal areas (Table 1).

Regarding non-epileptiform abnormalities, interesting evidence is given by the use of EEG band power analysis.

**Figure 1:**
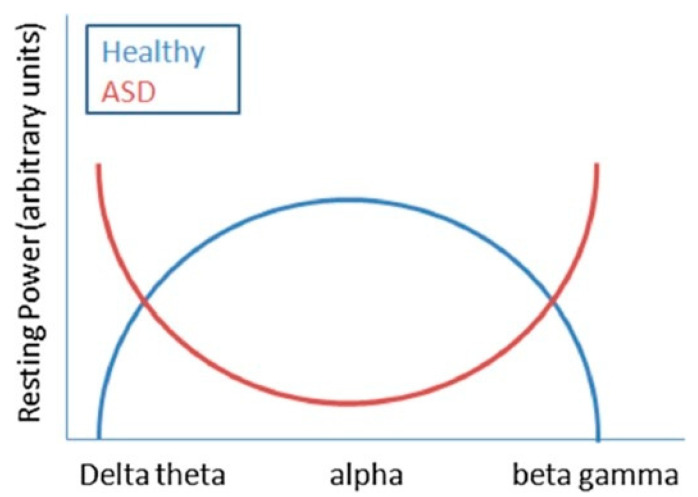
Illustration of the U-shaped profile of abnormal power pattern in autism spectrum disorders (from Wang et al., 2013) [49].

The clinically relevant frequency bands of the EEG range from 0.3 to 100 Hz. The main frequency bands are Delta (1 to 3 Hz), theta (4 to 7 Hz), alpha (8 to 12 Hz), beta (13 at 35 Hz), and gamma (>35 Hz) [49]. Delta waves dominate deep sleep. Theta waves occur during stages 1 and 2 of the sleep. The alpha rhythm is typical of mentally inactive state with closed eyes and psychosensory rest. Beta waves occur in all healthy subjects, with open eyes and associated with physiological activation, attention, concentration, analytical thinking, and in states of particular mental commitment or motor tasks. Finally, gamma waves are associated with working-memory tasks and with a variety of early sensory responses [50]. The resting EEG in patients with ASD shows an increased activity of delta, theta, beta, and gamma spectral bands, with reduced activity of medium frequencies (alpha) averaging with Fast Fourier Transform [39]. This abnormal power pattern describes a U-shaped profile (Figure 1), in which the activity of extreme frequencies (low and high) is significantly increased, while that of medium frequencies appears reduced [39,51].

However, contrasting data reveal that resting-state parietal alpha power has a positive association with some traits characteristic of the cognitive rigidity, that is, the difficulty to adapt to changes and the tendency towards repetitive behaviors (evaluated with Broad Autism Phenotype Questionnaire—BAP-Q—*p* < 0.05) found in autism, suggesting that the broader autism phenotype could be associated with different EEG patterns [52].

Furthermore, in a cohort study of 19 children with ASD, a remarkable increase in gamma activity was evidenced compared to the control group (*p* = 0.002), mainly in frontal, parietal, and temporal regions [53]. Although the role of spectral power changes, in the developmental windows of children with autism, it is still not entirely clear; it could be correlated with cognitive and behavioral dysfunctions [54]. For instance, a recent longitudinal study has demonstrated that among high-risk toddlers for ASD, an increased frontal gamma activity is highly correlated with a reduced expressive language ability assessed through Mullen Scales of Early Learning (*p* = 0.007) [55].

The U-shaped profile could be partly attributed to an abnormal functioning of the GABAergic tone in the inhibitory circuit, which influences the development of plasticity and brain function and is thought to intervene in the modulation of EEG frequency bands [56]. Such anomalies could therefore be the result of a complex pattern of neurochemical alterations affecting the physiology of the interneuron inhibitory of the GABAergic system, intervening in the modulation of the excitatory activity of the pyramidal cells. Alterations of gamma activity are not a specific trait of patients with ASD but rather they are related to dysfunctions in the GABAergic system, observed in different developmental disorders, such as schizophrenia and epilepsy [57].

However, there is evidence that the development and connection of GABAergic interneurons are interrupted in the frontal and temporal cortex of patients with ASD [58] leading to an excitatory/inhibitory imbalance [59]. Moreover, in patients with ASD, the minicolumns in layer III of the neocortex are increased in number but more “narrow”, due to a reduction in the dendritic space [60], and this anomalous organization of minicolumns could determine a deficit of fibers and GABAergic tone [61].

In some cases, the increased activity of low frequencies could be a compensatory mechanism in patients with ASD to stop the activation of high excitatory frequencies produced by GABAergic system dysfunction [62]. The disrupted inhibitory control system could be linked to increased levels of inattention and impulsivity detected in patients with ASD [63].

Abnormal power patterns seem to occur very early in toddlers. A recent study has analyzed 129 neonatal EEG finding a significant association between higher frequency EEG power (primarily in right frontal, polar, left parietal and right temporal brain regions) and socioemotional competences evaluated through the brief infant toddler social emotional assessment (BITSEA) [64].

A study analyzed EEG power taken across multiple developmental windows (3–12 months, 12–24 months, and 3–36 months) and spatial configurations, and it indicated that delta and gamma frequency power trajectories can consistently differentiate children with ASD from others especially frontal EEG power in the first year after birth [65,66].

However, a recent review about EEG frequency bands in resting state showed no significant or poor difference in almost all bands except for delta and beta eyes closed and alpha eyes open bands. Nevertheless, since methodological challenges and limitations relating to frequency band analysis literature studies have poor consistency, no general pattern can be individuated [67].

In addition to the spectral differences detected in patients with ASD, variations have also been reported in the cerebral hemisphere asymmetry. Most of the data in the literature regarding resting EEG show a greater activity (power and/or amplitude) in all frequency bands in the left hemisphere compared to the right hemisphere in individuals with ASD [68,69,70].

In order to identify a possible autistic precocious biomarker, a lot of studies used quantitative EEG for the diagnosis of autism. The most significant finding in this field is a decreased inter- and intra-hemispheric coherence in subjects with autism compared to healthy controls. The coherence is the correlation between two EEG signals in a specific frequency band, and it is considered a measure of connectivity. People with autism particularly have lower delta and theta coherence with short-medium inter-electrode distances and lower delta coherence with the long interelectrode distances [71,72].

## 4. Treatment Implications in Patients with ASD and SEAs

Despite the limitations of the above-mentioned studies, evidence indicates that the presence of EEG abnormalities is correlated to cognitive and behavioral impairment in children with autism and suggests worse developmental outcomes.

The decision to start a treatment of SEAs in seizure-free patients should be based on two assumptions. First, SEAs directly cause cognitive and behavioral impairment in children with ASD.

Second, treating SEAs could prevent the onset of seizures.

However, what is the predictive value of EEG abnormalities in the development of later epilepsy remains an open question. In a study, Parmeggiani et al. analyzed a large sample of 345 subjects with autism where EEG SEAs without clinical seizures are present in 81 (23.5%) cases. Isolated EEG abnormalities preceded epilepsy onset only in 8 cases (9.3%). EEG abnormalities are mainly present in childhood (*p* < 0.05) (peak from 5 to 10 years) while epilepsy tends to occur significantly (*p* < 0.001) later (peak from 20 to 25 years) [8].

Another study performed by Kanemura and colleagues had similar results. They followed 21 children (between the ages of 3 and 6 years) with ASD over a 6-year period and documented SEAs in 52.4%. Six of these patients (28.6%) developed epilepsy. The presence of frontal epileptiform abnormalities was significantly associated with later development of epilepsy compared to centrotemporal paroxysms (*p* < 0.003) [73].

A similar association has been found in a study that analyzed 16 children with high functioning autism in which only one patient with abnormal frontal fast activity developed seizures later in time [74].

The current clinical experience in autistic patients suggested that a large percentage of these patients could develop seizures in adolescence or adulthood [75]. However, the small number of longitudinal studies does not allow to clearly establish the predictive value of SEAs for subsequent epilepsy. Therefore, pharmacological treatment of SEAs in order to prevent epilepsy is to date not supported by evidence.

Similarly, the hypothesis of treating SEAs without clinical evidence of seizures in order to improve autism core symptoms or neuropsychological performances is not clearly supported by sufficient evidence. The rationale for SEAs treatment is that they could interfere with normal neural functioning causing deleterious transient cognitive impairment [19,76].

There is some evidence that subclinical EEG discharges can cause transitory cognitive impairments and that a pharmacological treatment leads to a reduced discharge rate and to an improved global rating of psychosocial functions in these patients [77].

Valproic acid (VPA) and derivatives are the most used drugs in the treatment of EEG abnormalities. Literature data about utilization of VPA in the treatment of SEAs in seizures-free children confirmed significant improvements in language and social skills [78,79].

The study by Chez et al. on 176 patients treated with VPA (drug levels 80–120 mg/dL) has shown that SEAs improve or normalize 63.6% of them (46.6% normalization and 17% improvement) [30].

Moreover, a retrospective pilot study on 14 patients with ASD showed improvements in autism core symptoms especially in affective instability, impulsivity, and aggression after treatment with divalproex sodium (mean dose: 768 mg/day, range: 125–2500 mg/day) [80].

Other studies demonstrated that administration of lamotrigine (LTG), an antiepileptic also used in psychiatry for bipolar treatment, provokes the reduction of electroencephalographic discharges and a concomitant improvement in behavior, alert, concentration, and general performances. Remarkably, improvements occur only in patients showing a significant reduction in frequency (*p* < 0.05) or duration (*p* < 0.05) of discharges [81,82].

VPA and LTG are often used as mood stabilizers. Therefore, behavioral improvements could be attributed to the direct effect of these substances rather than SEAs reduction.

However, similar results have been found with the administration of levetiracetam (LVT), which is not traditionally considered a mood stabilizer. In a blinded, prospective, and randomized controlled trial, the administration of LVT (at a dosage of 60 mg/kg/day) in patients with ASD and EEG abnormalities in absence of seizures has determined a reduction of SEAs in 75% of cases. Particularly, behavioral and cognitive functions at the 6-month follow-up, assessed using Psychoeducational Profile—third edition (PEP-3; Chinese version), Childhood Autism Rating Scale (CARS), and Autism Behavior Checklist (ABC), were significantly improved (*p* < 0.05), compared to baseline evaluation [83].

There are also studies reporting improvements of clinical functions and neurophysiological skills in seizure-free patients with SEAs treated with corticosteroids [84]. A retrospective study on 20 steroid-treated patients with ASD showed a significant improvement (*p* = 0.00001) in language function and a significant reduction (*p* = 0.00001) in DSM-IV ASD scaled symptoms. However, no significant reduction across time of SEAs was detected, and almost all the patients had side effects such as Cushingoid appearance, weight gain, and enhanced appetite [85].

Finally, the use of neurosurgery in patients with autism and epileptiform EEGs without seizures is very controversial. To our knowledge, there is only one study that has collected 11 patients who underwent surgery to control the epileptiform activity. Patients after neurosurgery showed mild to moderate improvements in language and autistic features [86].

Waiting for controlled double-blinded studies proving clinical improvements of autistic symptoms following antiepileptic drug administration, the pharmacological therapy to treat EEG abnormalities in absence of seizure remains controversial. Definitively, to treat or not to treat SEAs remains an open question because there is no solid evidence for or against treatment of SEAs. However, SEAs in asymptomatic individuals should never be treated. However, if there are cognitive dysfunctions, regression, or neurological symptoms, a pharmacological therapy should be considered [87].

## 5. Conclusions

Despite the long-established association between ASD and SEAs, to date there is little strong evidence about the implication of this finding. The main lack of evidence concerns the role of SEAs in the complex etiopathogenesis of autism and if they can be considered a good biomarker or a therapeutic target.

Literature data suggest a widely varying frequency of SEAs in patients with ASD, which is anyway significantly higher than in the general population. The most common site of abnormal electrical discharges seems to be the right temporal lobe and SEAs appear independent of age and gender, but they are more frequently associated with lower intellectual functioning, more serious dysfunctional behaviors, and they are often sign of worse prognosis, probably because they reflect a severer brain dysfunction disrupting neuronal excitability.

The broader autism phenotype is associated with different EEG epileptiform and non-epileptiform abnormalities. However, an increased activity of delta, theta, beta, and gamma bands, with reduced activity of alpha frequencies, is a frequent pattern observed in patients with ASD. This EEG pattern is probably the expression of an imbalance of inhibitory GABAergic interneurons. Another recently proposed EEG pattern is a decreased inter- and intra-hemispheric coherence in subjects with autism compared to healthy controls.

However, literature data, to date, are not able to find a causal relationship between SEAs and the subtypes of ASD phenotype or to exclude that they are simply an epiphenomenon of the neurochemical alterations in autism etiology.

To date, there is little consensus or conclusive evidence about the role of SEAs in determining the complex ASD phenotype. Hopefully, more cohesive practice and collaboration between researchers should lead to more conclusive results.

The lack of evidence is even greater for what concerns the treatment of SEAs in seizures-free patients with autism. Some literature data showed an improvement of autism core symptoms and neurophysiological skills in patients with SEAs and ASD after therapy with antiepileptic drugs. However, in absence of controlled double-blinded studies, it is impossible, to date, to give a final evaluation about the effectiveness and appropriateness of pharmacological treatment of SEAs.

To give adequate answers about the characteristics of EEG abnormalities in ASD and about their related therapeutic implications, further quality investigations are needed in order to provide appropriate guidelines to clinical practice.

## Figures and Tables

**Table 1:** Subclinical Electroencephalographic Abnormalities (SEAs) in children with Autism spectrum disorder (ASD) without clinical seizures.

	Subjects	Mean Age (Years)	% SEAs	EEG Findings	Other Findings
Rossi et al. (1995) [29]	106	12.5	18.9%	Focal SEAs were mainly localized on centro-parieto-temporal regions	Higher incidence of severe intellectual disability
Tuchman and Rapin (1997) [30]	335	Not reported	8%	Centrotemporal SEAs (~50%)	Higher incidence of Regression
Hrdlicka et al. (2004) [31]	77	9.1 ± 5.3	48%	Non-epileptiform abnormality (18%); Epileptiform discharges (30%)	Abnormal early development
Canitano et al. (2005) [32]	46	7.8 ± 2.7	22%	Focal 50%; Multifocal 50%; Diffuse 20%	No difference in regression rate
Chez et al. (2006) [33]	889	5.3	60.7%	Right temporal region (21.5%), bilateral temporal central spikes (20.2%). Generalized spike–wave activity (16.2%) left temporal activity (15.2%)	No difference in regression rate
Hara (2007) [34]	97	18–35	21%	Temporal region SEAs (60%)	Lower IQ and social skills in the epileptics rather than in patients with SEAs
Baird (2006) [35]	64	3 ± 0.7	~31%	Not specified	No difference in regression rate
Akshoomoff et al. (2007) [23]	60	2–6	~32%	78% epileptiform abnormalities and 22% focal slowing	SEAs are more frequent in low-functioning autism (72%)
Hartley et al. (2010) [36]	123	Not reported	31%	No significant abnormal discharge location	No differences in behavior between children with ASD with or without SEAs
Yasuhara (2010) [6]	1014	9.3 ± 3.4	~49%	SEAs in frontal lobe (65.6%)	Lower IQ in patient with SEAs
Parmeggiani et al. (2010) [8]	345	mean age 10.5 years	23.5%	SEAs in temporal and central areas (31.4%).	SEAs were not related to autistic regression
Valvo et al. (2013) [28]	206	7.1 ± 3.8	51%	EEG abnormalities were not specified	Tall stature was significantly associated with SEAs
Mulligan and Trauner (2014) [24]	101	7.06 ± 3.74	50%	The most frequent location was frontal with multifocal or generalized epileptiform abnormalities	Higher incidence of SEAs in children with stereotypies and aggressive behavior
Swatzyna et al. (2017) [27]	140	4–25	36%	EEG abnormalities were not specified	SEAs are regardless of age or gender
Capal et al. (2018) [37]	372	45 ± 16.4 (months at ASD diagnosis)	25.5%	67.4% epileptiform, 36.8% other abnormalities. 83% Focal and more frequent in the left temporal region	Impaired adaptive functioning
Milovanovic et al. (2019) [38]	112	6.58 ± 3.72	12.5%	Slow background activity, abnormal sleep architecture	Better motor skills in patient without SEAs
Nicotera et al. (2019) [39]	69	6.5 ± 4.01	39.13%	Non-epileptiform abnormalities 13%; epileptiform abnormalities 26%	SEAs significantly higher in patients with severer forms of ASD.
